# The Early Developmental Outcomes of Prenatal Alcohol Exposure: A Review

**DOI:** 10.3389/fneur.2018.01108

**Published:** 2018-12-18

**Authors:** Sivenesi Subramoney, Emma Eastman, Colleen Adnams, Dan J. Stein, Kirsten A. Donald

**Affiliations:** ^1^Division of Developmental Pediatrics, Department of Pediatrics, and Child Health, Red Cross War Memorial Children's Hospital, University of Cape Town, Cape Town, South Africa; ^2^Department of Psychiatry and Mental Health, University of Cape Town, Cape Town, South Africa; ^3^Unit on Risk and Resilience in Mental Disorders, South African Medical Research Council (SAMRC), Cape Town, South Africa

**Keywords:** early childhood, developmental outcomes, fetal alcohol spectrum disorders, prenatal alcohol exposure, neurodevelopment

## Abstract

**Aim:** This paper systematically reviews the literature on the effects of prenatal alcohol exposure on early child development from birth to 5 years with the aim to synthesize the developmental outcomes associated with prenatal alcohol exposure, and inform further research to improve our knowledge of the manifestations of prenatal alcohol exposure.

**Methods:** Electronic databases (MEDLINE, Psych INFO, and Psych ARTICLES) were searched to find papers on the developmental outcomes of prenatal alcohol exposure in neonates, infants and toddlers and pre-school aged children. Studies were selected based on participants self-reporting alcohol consumption during pregnancy (either prospectively or retrospectively) and/or children being diagnosed with FASD based on a standardized assessment that includes a dysmorphology examination. The search was limited to peer-reviewed, English language studies involving human subjects, up to 5.5 years old.

**Results:** Out of the 1,684 titles screened, a total of 71 papers were identified as relevant and included in this review. The majority of studies were prospective longitudinal studies. A range of assessment modalities (or tools) was used to determine neurodevelopmental outcomes of prenatal exposure to alcohol in the age group under review, the most frequently described being the Bayley Scales of Infant and Toddler Development (BSID) (*n* = 19). Studies varied in terms of the dose, frequency, and timing of alcohol consumption during pregnancy and methodology used to assess alcohol consumption. Findings demonstrate extensive evidence for poor global developmental outcomes in children prenatally exposed to alcohol, particularly with moderate to severe levels of prenatal alcohol exposure.

**Conclusion:** The outcomes related to lower levels of prenatal alcohol exposure as well as outcomes in specific developmental domains, are poorly understood. Further research should aim to clarify the more subtle or less easily measurable manifestations of prenatal alcohol exposure on early development when the potential for greatest impact of interventions is highest.

Drinking of alcohol during pregnancy is known to have widespread negative effects on fetal growth and development (1–4). The term “Fetal alcohol spectrum disorder” (FASD) encompasses the broad spectrum of effects associated with prenatal alcohol exposure. Deficits include severe growth delay, facial dysmorphology, and cognitive and behavioral impairment (i.e., fetal alcohol syndrome; FAS) as well as the presence of cognitive or behavioral deficits without the presence of facial dysmorphology (i.e., alcohol-related neurodevelopmental disorder; ARND) (4, 5). Global estimates of prevalence range from 2 to 5% of births for FASD (6–9), with rates as high as 18 to 26% in certain regions in South Africa (6, 10–13). FASDs thus present a serious public health and economic burden (14, 15) and are recognized as a major cause of developmental disability and loss of developmental potential in children (16).

A growing body of literature documents poor global developmental outcomes (4, 17, 18), as well as domain specific deficits in motor, learning, attention, processing speed, language, and executive functioning (19–21) amongst children with prenatal alcohol exposure. Previous research has also highlighted that the severity and type of impact associated with prenatal alcohol exposure depends on several factors, including but not only related to metrics of alcohol exposure. These factors include the quantity, frequency, and timing of alcohol consumption during pregnancy (21–24), maternal risk factors (25–27), and genetic predispositions (28). In addition, postnatal socioeconomic and environmental influences may compound the outcomes related to prenatal alcohol exposure (29, 30). Due to the above mentioned influencing factors as well as variations in study design and methodologies, there are currently many knowledge gaps on the outcomes associated with prenatal alcohol exposure and research has yet to establish whether FASDs exhibit a unique neurocognitive profile (31).

FASDs are difficult to diagnose in early childhood and as a result the manifestations of prenatal alcohol exposure during early childhood are particularly poorly understood (32). There remain inconsistencies in what is known regarding the early developmental outcomes related to maternal alcohol consumption during pregnancy. Namely, findings on the dose specific effects of alcohol exposure are not clear (33, 34) and there is a lack of clarity on the manifestations of outcomes associated with prenatal alcohol exposure at specific stages during early development. The early developmental years are a unique period where trajectories are being established and children are most likely to benefit from interventions (35), there is thus a crucial need for research that broadens current knowledge on the early developmental outcomes related to prenatal alcohol exposure.

Recent reviews on prenatal alcohol exposure primarily focus on the effects of varying quantities of prenatal alcohol exposure (21, 36) or the effects of prenatal alcohol exposure on specific outcomes (33) throughout childhood (37). There has however been little focus on synthesizing the developmental manifestations of prenatal alcohol exposure across the duration of the early childhood period. This paper aims to systematically review the literature on the effects of prenatal alcohol exposure on developmental outcomes during early childhood. Consolidating the existing literature on the effects of prenatal alcohol exposure during infancy and early childhood helps provide clarity on our current understanding on prenatal alcohol exposure and provides directions for future research.

## Methods

We conducted an electronic search through the following databases: MEDLINE, Psych INFO, and Psych ARTICLES. An inclusive search using the following keywords were used to identify relevant studies: “prenatal alcohol exposure,” “fetal alcohol exposure,” “fetal alcohol spectrum disorders,” “fetal alcohol syndrome,” “FAS,” “FASD,” “maternal alcohol consumption,” and “maternal drinking.” The search was not restricted to starting limits and was extended to 31 October 2017. The search was restricted to include case-control or follow-up studies including human subjects, up to 5.5 years old, published in peer-reviewed, English language journals. Case studies, case reports, reviews, and studies that fell out of our age range were excluded. Additional studies were identified through the reference lists of studies identified in the initial database search. Developmental outcomes were broadly defined to include global, cognitive, motor, behavior, socio-emotional, language, executive functions, information processing, and attentional domains. Studies were selected based on participants self-reporting alcohol consumption during pregnancy (either prospectively or retrospectively) or children being diagnosed with FASD.

Given that heterogeneities in methodology and outcome measures used amongst studies makes quantitative comparisons between studies challenging, we chose a systematic qualitative approach for this review. Furthermore, since this review aims to address and synthesize the broad range developmental outcomes associated with prenatal alcohol exposure, we found a narrative approach more appropriate than a meta-analytic approach that requires more stringent study selection criteria.

## Results

**Figure 1:**
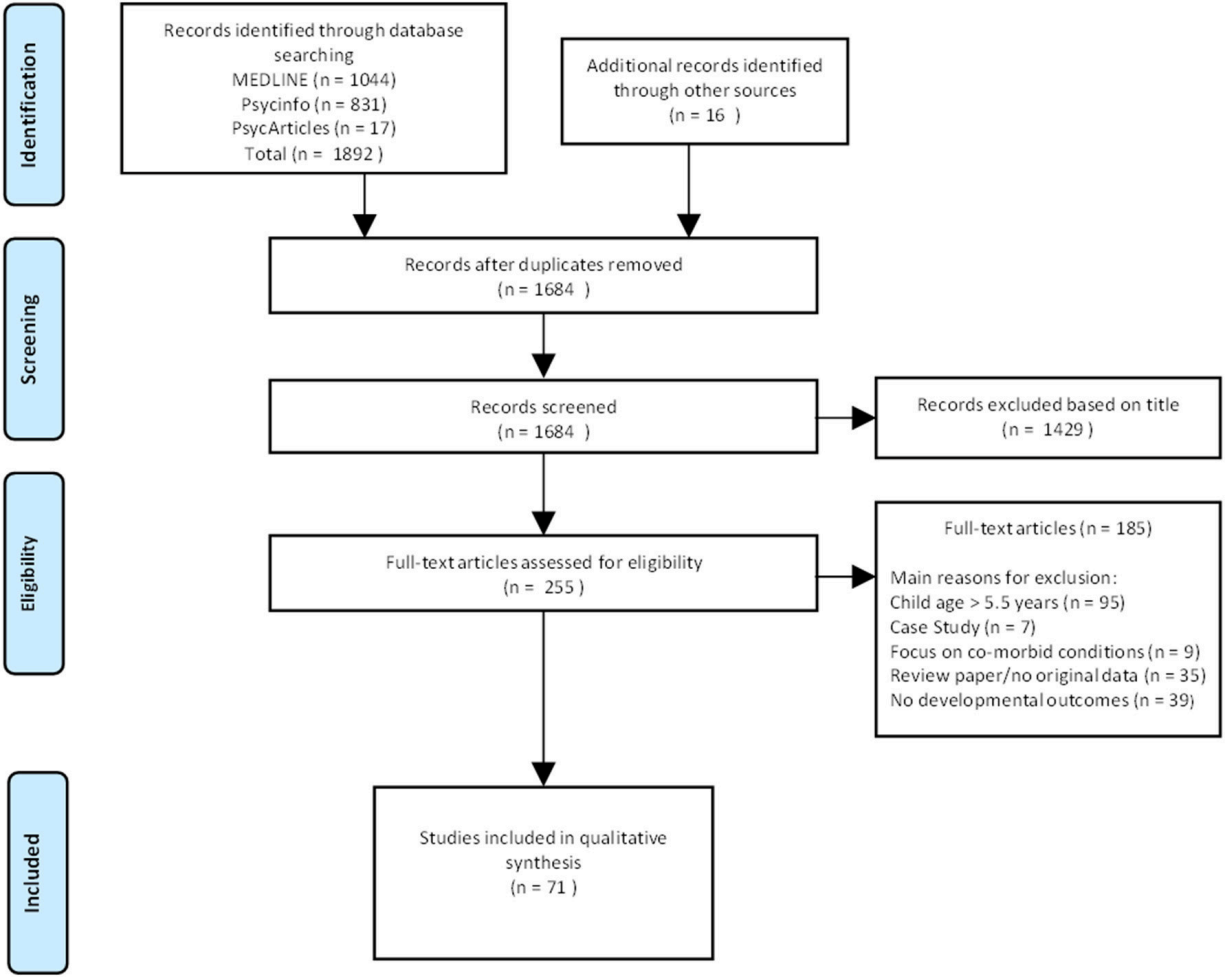
Diagram of study selection process.

Seventy one papers met our criteria and were included in this review (see Figure 1). Selected studies are listed in Table 1. The majority of studies (*n* = 57) drew samples from prospective longitudinal cohorts. Most studies were conducted in the United States of America (USA, *n* = 34). Other publications included various countries within Europe (*n* = 20), Canada (*n* = 10), South Africa, (*n* = 5), and Australia (*n* = 2). Generally, studies compared children with prenatal alcohol exposure with a control group (either abstainers or light drinkers) with less focus on children with an established FASD diagnosis. Only 7 studies focused on children diagnosed with FASDs (17, 29, 53, 66, 81, 102, 103), negative developmental outcomes associated with prenatal alcohol exposure were evident in all 7 studies. With the exception of nationally-representative population-based studies (24, 49, 71, 75, 78, 82, 95, 97, 101, 104–107) and a few studies that recruited exclusively middle-income participants (43–45), most samples were recruited from clinics that served low socioeconomic status (SES) communities. Negative effects of prenatal alcohol exposure were more apparent in studies conducted in low income countries (17, 29, 67) than studies conducted in high income countries (24, 75, 78, 101, 108). Studies ranged in the pattern of alcohol exposure including low to moderate levels of exposure (75), isolated binge drinking (82, 95), frequent heavy drinking (46) as well as the duration of exposure including early (49) and prolonged (72) exposure.

**Table 1:** Studies examining developmental outcomes in young children with prenatal alcohol exposure.

**Author/Year**	**Setting**	**N**	**Age**	**Alcohol consumption**		**Tests**	**Outcomes assessed**	**Key findings**
				**Control**	**PAE**			
**NEONATES (< 1 MONTH)**								
Staisey and Fried (38)	Ottawa, Canada	59	1 day; 1 m	Abstainers/ Light drinkers (0.01 - 0.13 Oz AA/day)	Oz AA/day 0.14–0.85; >0.85	Prechtl neurological examination	Motor Behavior	Decreased muscle tone and increased startle reaction. Findings no longer evident at 30 days
Smith, et al. (39)	Atlanta, USA	149	3 days	Abstainers	Oz AA/week 1st and 2nd trimester exposure (*M* = 10.7); 1st−3rd trimester exposure (>33)	BNBAS	Motor Behavior	Main effects for dose and duration of alcohol exposure. Drinking throughout pregnancy related to decreased orientation and autonomic regulation
Coles et al. (40)	Atlanta, USA	103	3 days; 1 m; 6 m	Abstainers	Oz AA/week 1st and 2nd trimester exposure (*M* = 14.14); 1st−3rd trimester exposure (*M* = 12.18)	BNBAS; BSID	Motor Behavior	Drinking throughout pregnancy related to poorer orientation and motor performance. Findings persisted at 30 days. Drinking throughout pregnancy related to lower MDI and PDI scores at 6 months
Coles et al. (41)	Atlanta, USA	31	3 days; 14 days; 30 days	Abstainers	Oz AA/week 1st and 2nd trimester exposure (*M* = 16.25); 1st−3rd trimester exposure (*M* = 13.34)	BNBAS	Motor Behavior	Poorer motor performance, autonomic regulation, and abnormal reflexes amongst neonates with longer duration of prenatal alcohol exposure
Oberlander et al. (42)	Cape Town, RSA	28	3 days	Abstainers/light drinkers (0.5 Oz AA/day)	>14 drinks/week or 1 binge per month	BNBAS	Motor Behavior	Less arousal on the BNAS scale in exposed group (non-significant)
Fried et al. (43)	Ottawa, Canada	250	9 days	Non- /infrequent drinkers (0–0.13 Oz AA/day)	Oz AA/day 0.14–0.85; >0.85	Prechtl neurological examination; BNBAS	Motor Behavior	Relatively low levels of prenatal alcohol exposure associated with decreased arousal at 9 days
Fried and Makin (44)	Ottawa, Canada	250	9 days	Non- /infrequent drinkers (0–0.13 Oz AA/day)	Oz AA/day 0.14–0.85; >0.85	BNBAS	Motor Behavior	Relatively low levels of prenatal alcohol exposure associated with increased irritability at 9 days
Streissguth et al. (45)	Seattle, USA	417	9 days; 1 m	Abstainers	Oz AA/day 0.01–0.10; 0.11–0.99; 1.00–1.99; 2.00 or more	BNBAS	Motor Behavior	Alcohol consumption during pregnancy is related to lower arousal and poorer habituation in newborn infants
**INFANTS (2–23 MONTHS)**								
Autti-Rämö and Granström (46)	Helsinki, Finland	80	4; 6; 12 m	Abstainers/ light drinkers (< 28 g/week)	g/week (>28 g); 1st trimester only; 1st−2nd trimester; 1st−3rd trimester	MFDD	Global	Increased duration of maternal alcohol consumption associated with poorer motor scores at 6 months and motor and cognitive scores at 12 months. Occurrence of developmental delay increased over year
Ioffe and Chernick (47)	Winnipeg, Canada	38	4; 48 m	Light drinkers (< 15 ml AA /occasion, < once/ month)	15–60 ml AA >once/month; >60 ml AA >twice/ month; alcoholic mothers	BSID; MSCA	Global	MDI and PDI scores lower in the alcohol exposed infants at 4 m. Poorer performance on MSCA at 4 years
Lemola, et al. (48)	Switzerland	458	5; 17 m	No risk drinking (< 3 on AUDIT)	>3 on AUDIT	IBQ	Behavior	Prenatal alcohol exposure associated with increased irritability at 5 months
Alvik et al. (49)	Oslo, Norway	1303	6 m	Abstainers	≥ 5 drinks/occasion Once/week; >Once/week	DTS; ITSC; ASQ	Behavior	Binge drinking once a week predicted difficult temperament.
Kable and Coles (50)	Atlanta, USA	118	6 m	< 7 MSAC	≥7 MSAC	FTII; auditory stimuli	Information processing Attention	Alcohol exposure group show slower information processing and attention regulation
Coles et al. (22)	Western Ukraine	367	6 m	Abstainers	Oz AA/day 0.87 to 5.15	BSID-II; BRS	Global Behavior	Peri-conception alcohol exposure related to lower MDI scores and poorer total behavioral rating scores
Coles et al. (51)	Atlanta, USA	70	6; 12m	< 7 MSAC	≥7 MSAC	BSID-II; BRS	Global Behavior	Prenatal alcohol exposure had lower language scores at 6 m, lower cognitive facet scores at 12m and greater number of developmental delays
Fraser et al. (52)	Nunavik, Quebec, Canada	216	6 m	0	Oz AA/day Consumed alcohol during pregnancy (*M* = 0.1) Occasional binge drinking (M = 4.8)	TAC; FTII	Information processing	No effects of occasional binge drinking on information processing
Molteno et al. (53)	Cape Town, RSA	85	6.5; 13; 60 m	Unexposed (< 0.5 Oz AA/day)	Oz AA/day >1.0/>2 binge drinking episodes during 1st trimester/pFAS /FAS	ADBB	Behavior Socio-emotional	Prenatal alcohol exposure associated with increased infant emotional withdrawal and decreased activity. Children diagnosed as FAS and pFAS at 5 years had greater emotional withdrawal as infants
Jacobson et al. (54)	Detroit, USA	103	6.5 m	Abstainers/ light drinkers (0.01–0.49 Oz AA/day)	Oz AA/day 0.5–0.99; 1.00–1.99	RT task	Reaction time	Lower levels of exposure associated with longer RT
Jacobson et al. (55)	Detroit, USA	403	6.5; 12; 13 m	Abstainers	Oz AA/day 0.01–0.49 0.5–0.99 1.00–1.99 >2.00	FTII; Symbolic task; Piagetian task; BSID; TAC	Information processing Attention	Dose dependent effects of prenatal alcohol exposure on information processing at 6 months, sustained attention at 12 months. Mean sustained directed attention negatively associated with McCall Index scores (obtained from BSID)
Haley et al. (56)	New Mexico, USA	55	5–7 m	Low frequency (1–2 drinks/week)	>2 drinks/week	SFP	Socio-emotional	Significant effect of alcohol exposure on infant affect
Davies et al. (17)	De Aar, RSA	392	7–12 m; 17 −21 m	Healthy controls	pFAS/FAS	GMDS	Global	FASD group had lower total developments scores, with marked motor delays. Greater differences with FASD and non-FASD groups over time
Davies et al. (29)	De Aar, RSA	121	7–12 m; 60m	Healthy controls	pFAS/FAS	GMDS	Global	Griffith total score higher for controls than the FAS/pFAS group. FAS/pFAS trajectories declined more than controls for eye-hand, performance and total scores at 5 years
Streissguth et al. (57)	Seattle, USA	462	8 m	< 0.1 Oz AA/day	Oz AA/day < 1.00; ≥1.00; ≥2.00	BSID	Global	Alcohol exposure predicts MDI and PDI scores
Richardson et al. (58)	Pittsburgh, USA	193	9; 19 m	Abstainers	Drinks/day >1 during 1st trimester; 1> during 1st−3rd trimester	BSID	Global	No effects of prenatal alcohol exposure on BSID
Brown et al. (59)	USA	300	9 m	Abstainers/light drinkers (< 1 drink/ week)	Drinks/week 1–3; >4	BSF-R; NCATS; ITSC	Global Behavior	Dosage related to sensory regulation, MDI, and PDI scores. Significant difference with undesirable social engagement and child interaction (1–3 drinks/week) and passive behavior (>4 drinks/week
Jirikowic et al. (60)	Seattle, USA	18	6–15 m (*M* = 10.7 m)	No/low prenatal alcohol exposure	F-BAS score ≥4; ≥24	SFP; IBQ; ITSC	Behavior	Fewer social monitoring behaviors amongst infants with prenatal alcohol exposure
Greene et al. (61)	Cleveland, USA	260	12; 24; 36; 58 m	MAST scores < 5	MAST scores ≥5	BSID (MDI); SB; WPSSI	Cognitive	No effects of prenatal alcohol exposure on cognitive scores
O'Connor and Brill (62)	Los Angeles, USA	26	12 m	Light drinking (< 3 drinks/occasion)	>3 drinks/occasion	BSID (MDI)	Cognitive	Lower MDI scores in the moderate alcohol exposure than the light drinking group
O'Connor et al. (63)	Los Angeles, USA	44	1 y	Light drinking (≤ 0.10 Oz AA/day/ ≤ 2 drinks/occasion)	>0.10 Oz AA/day/ >2 drinks/occasion	*MCRS*; BSID (MDI)	Cognitive Behavior	Infants with prenatal alcohol exposure had significantly lower MDI scores and greater irritability than infants whose mothers abstained from drinking
Seagull et al. (64)	Detroit, USA	120	1 y	Abstainers	Drinks/day ≤ 1; >1; >5	BSID	Global	Lower MDI but similar PDI scores infants with prenatal alcohol exposure (regardless of amount)
Fried and Watkinson (65)	Ottawa, Canada	126	1 y; 24 m	None/light (< 0.14 Oz AA/day)	> 0.85 Oz AA/day	BSID; HOME; IBR; NRDLS	Global Behavior Language	No associations between alcohol exposure and MDI, PDI outcomes at 1 year. Moderate levels of alcohol exposure associated with lower MDI scores and poorer comprehension at 2 years
Golden et al. (66)	USA	24	12 m	Healthy controls	Maternal substance abuse during pregnancy/FASD	BSID	Global	Prenatal alcohol exposure group had lower MDI and PDI scores than the control group. Prenatal alcohol exposure group classified as developmentally delayed
Molteno et al. (67)	Cape Town, RSA	107	13 m	Light drinking and abstainers (0.1 Oz AA/day)	1 >Oz AA/day	Symbolic play task; JSAIS	Cognitive	Prenatal alcohol exposure related to poorer elicited play performance and predicted 5 year digit span
Gusella and Fried (68)	Ottawa, Canada	84	13 m		(*M* = 0.24 Oz AA/day during first trimester	BSID	Global	Maternal alcohol consumption associated with poorer Bayley mental scale performance, decrease in spoken language and verbal comprehension
Jacobson et al. (69)	Detroit, USA	382	13 m	Abstainers/light drinkers (0.01–0.24 Oz AA/day	Oz AA/day 0.25–0.49; 0.50–0.99; 1.00–1.99; >2.00	BSID	Global	Second and third trimester drinking lead to poorer outcomes. Specific deficits related to imitating modeled behavior, standing and walking
Forrest et al. (70)	Dundee, UK	592	18 m	Light drinkers (1–49 g AA/week)	g/week 50–99; ≥100	BSID	Global	No effect of prenatal alcohol exposure on PDI and MDI. Psychomotor scores increased with high alcohol consumption
Parry and Ogston (71)	Dundee, Odense, Berlin	592 247 522	18 m	Abstainers	g/week >0–29; 30–59; 60–89; 90–119; >120	BSID; BRS	Global Behavior	No relationship between prenatal alcohol exposure and MDI, PDI, and responsiveness scores.
Autti-Rämö and Granstrom (72)	Helsinki, Finland	109	18 m	Abstainers to light drinkers (< 28 g per week).	g/week (>28 g); 1st trimester only; 1st−2nd trimester; 1st−3rd trimester	Developmental assessment developed by MLG	Global	Long exposure groups had poorer performance in motor and language than non-exposed group. Increased cognitive delay when compared to performance at 12 months
Larsson et al (73)	Stockholm, Sweden	80	20 m	Controls (average less than 30 g AA/day)	ml AA 15–60 ml AA >once/month; >60 ml AA, >twice/month; alcoholic mothers	GMDS	Global	3 of 6 children with continuous heavy prenatal alcohol exposure showed characteristics of FASD. Continuous drinking group had lower personal/social, eye and hand coordination, performance, and behavior (hyperactivity, short attention span) scores than groups 1 and 2
Greene et al. (74)	Cleveland, USA	359	12; 24; 36 m	Abstainers	AA/day >0– < 1.00; >1.00– < 5.00; >5.00	SICD	Language	No relationship between alcohol exposure and language indices
**TODDLERS (25–47 MONTHS)**								
O'Leary et al. (24)	Western Australia	1739	24 m	Abstainers	g/occasion ≤ 20; 10– < 50; >50; >20–>50	ASQ	Language	Percentage of language delays highest for children with binge and moderate to heavy amounts of alcohol exposure. No effect of low exposure on language
Robinson et al. (75)	Western Australia	2370	24; 60m	Abstainers	Drinks/week < 1; 2–6; 7–10; >11	CBCL	Behavior	Light to moderate intake in the first 3 months was associated with CBCL scores indicative of positive behavior
Kaplan-Estrin et al. (76)	Detroit, USA	92	13 m, 26 m	Abstainers/Light drinkers (0–0.49 Oz AA/day)	0.50–6.50 Oz AA/day	BSID; CDIW; NCT; ELMS	Global language	PDI deficits seen at 1y 1m and 2y 2m. MDI analyses show spatial fine motor deficits associated with prenatal alcohol exposure. No effects of prenatal alcohol exposure on language
Autti-Rämö et al. (77)	Helsinki, Finland	108	27 m	0	g/week (>28 g); 1st trimester only; 1st−2nd trimester; 1st−3rd trimester	BSID; NRDLS	Global language	No effect on mental or language development in group whose mothers drank during the 1st trimester. Children whose mothers drank throughout pregnancy had poorer MDI and language scores than the group whose mothers drank during the first trimester
Faden and Graubard (78)	USA	8285	36 m	Abstainers	>1 drink/month; 1 drink /month 1–2 drinks/month Drinks/week 2; 3–5; 6–8; 9–13; 14–20; >21	DDST	Global	No effects of alcohol exposure on developmental indices. Greater behavioral problems associated with maternal drinking during pregnancy
Fried and Watkinson (79)	Ottawa, Canada	133	36 m	None/light (< 0.14 Oz AA/day)	>0.85 Oz AA/day	MSCA	Global	No effects of alcohol exposure on developmental scores at 48 months. Effects seen at 36 no longer significant
Olsen (80)	Odense, Denmark	251	42 m	Abstainers	Drinks/week 1–4; 5–9; >10	GMDS	Global	No association between maternal alcohol consumption and Griffith scores
Kalberg et al. (81)	New Mexico, USA	22	42 m (M)	Healthy controls	Children with prenatal alcohol exposure but without FAS; Children diagnosed with FAS	VABS	Motor	Children with FAS showed motor delays (FM>GM) and lower motor scores than children without prenatal alcohol exposure, and lower fine motor scores than both children without and with prenatal alcohol exposure (without FAS)
Sayal et al. (82)	UK	6355	47 m	< 4 drinks in a day	≥4 drinks in a day	SDQ	Behavior	Occasional binge exposure is associated with higher occurrence of behavioral problems in girls.
**PRESCHOOLERS (48-59 MONTHS)**								
McGee et al. (83)	San Diego, USA	51	48 m (*M*)	Abstainers	>4 drinks/occasion once per week/14 drinks per week during pregnancy	CELF-P	Language Cognitive	Alcohol exposed children had poorer language, effects no longer significant when IQ controlled
Barr et al. (84)	Seattle, USA	449	48 m	Light drinkers < 1.5 Oz AA/day	0.5–1.5 Oz AA/day	WFMSBSMS	Motor	Alcohol use during pregnancy related to reduced finger tapping count and TPT total time
Streissguth et al. (85)	Seattle, USA	452	48m	Abstainers	Oz AA/day 0.1–0.10; 0.11–1.00; >1.00	Vigilance task	Attention	Maternal alcohol used related to poor attention (more omission and commission errors) and longer reaction time
Boyd et al. (86)	Cleveland, USA	245	58 m	MAST < 5	MAST ≥ 5	CPT	Attention	No association between PAE and attention
Streissguth et al. (87)	Seattle, USA	421	48 m		>1.5 Oz AA/day	WPSSI	Cognitive	Prenatal alcohol exposure was associated with poor IQ
Noland et al. (88)	Ohio, USA	173	48 m	Unexposed (no evidence of alcohol exposure)	Exposed (positive urine and meconium screen)	TI task; Category fluency; MSCA; Finger sequencing task; WPSSI	Executive function Cognitive	TI performance lower in children with prenatal alcohol exposure
Chiodo et al. (89)	Detroit, USA	75	48 m	Light drinking (< 1.0 Oz AA/day)	>1.0 Oz AA/day	WPPSI; NTB; PBCL; FIST	Cognitive Behavior	Children with alcohol exposure showed greater deficits in full scale IQ score and arithmetic, symbol digit, and digit span scores, and greater problem behaviors on the PBCL
Landesman-Dwyer et al. (90)	Seattle, USA	272	48 m	Abstainers and occasional drinkers (*M* = 0.07 Oz AA/day)	*M* = 0.45 Oz AA/day	Naturalistic observation	Behavior	Children with moderate prenatal alcohol exposure less attentive, less compliant with parent commands, and more fidgety than children of non- and social drinkers
Larroque et al. (91)	Roubaix, France	155	54 m	Light drinkers (0– 1.49 Oz AA/day)	>1.5 Oz AA/day	MSCA	Global	Lower mean scores in the general cognitive index of the MSCA amongst those with prenatal alcohol exposure
O'Connor and Paley (92)	Los Angeles, USA	42	57 m		M 4.55 drinks (*SD* = 6.11)	PDS	Socio-emotional	Prenatal alcohol exposure was associated with greater negative affect
Fried and Watkinson (93)	Ottawa, Canada	135	60 m	None/light (< 0.14 Oz AA/day)	>0.85 Oz AA/day	MSCA; HOME	Global	No association between alcohol consumption and outcome variables
Bay et al. (94)	Denmark	685	60 m	Abstainers	Drinks/week 1–4; 5–9; ≥9	MABC	Motor	No association between low to moderate maternal alcohol intake during pregnancy and motor functioning
Kesmodel et al. (95)	Denmark	678	60 m	No binge drinking episode	> 5 drinks/ occasion	MABC	Motor	No systematic association between isolated episodes of binge drinking during early pregnancy and child motor function
Kesmodel et al. (96)	Denmark	1617	60 m	No binge drinking episode	>5 drinks/ occasion	WPSSI	Cognitive	No association between binge drinking and child intelligence
Falgreen Eriksen et al. (97)	Denmark	1628	60 m	Abstainers	Drinks/week 1–4; 5–9; ≥9	WPSSI	Cognitive	No difference in IQ scores between children whose mothers reported up to eight drinks per week during pregnancy compared to mothers who abstained. Children whose mothers reported drinking nine or more drinks per week had greater risk for low full scale and verbal scores
Skogerbø et al. (98)	Denmark	1628	60 m	Abstainers	Drinks/week 1–4; 5–9; ≥9 >5 drinks/occasion	SDQ	Behavior	No effects of low to moderate or binge drinking in early pregnancy on behavior.
Skogerbø et al. (99)	Denmark	1628	60 m	Abstainers	Drinks/week 1–4; 5–9; ≥9 >5 drinks/ occasion	BRIEF	Executive functions	No effects of low to moderate or binge drinking in early pregnancy on executive functions.
O' Connor et al. (100)	Los Angeles, USA	42	60 m	Abstainers	Drinks/occasion < 1 drink ≥2 drinks	Family interaction puzzle task; AQS	Behavior Socio-emotional	Prenatal alcohol exposure was related to attachment insecurity and predicted child negative affect
Alvik et al. (101)	Oslo, Norway	1116	66 m		Drinks/occasion >5>than once per month; >5>than once per week; >5 >once per week	SDQ	Behavior	Binge drinking predicted abnormal and borderline scores on the SDO. Binges (more than once per week) predicted high symptom scores) and hyperactivity
Fuglestad et al. (102)	Minneapolis, USA	100	36–66 m	Matched Controls	Diagnosed with FASD	EF Scale for early childhood; Delay of gratification task	Executive functions	Children with FASD performed poorly compared to normative data, those with FAS had largest deficits. IQ correlated with EF scale and delay of gratification task
Janzen et al. (103)	Saskatchewan, Canada	20	42–60 m	Matched controls	Diagnosed with FAS.	MSCA; Groove pegboard; Beery, TELD, CBCL	Global Behavior	FAS group displayed impaired visual-motor integration, greater frequency of behavioral problems, significant growth delays
Kilburn et al. (104)	Denmark	1333	60 m	Abstainers	Drinks/week 1–4; 5–9; ≥9 Binge drinking >5/occasion	Sternberg paradigm, WPSSI-R	Reaction time Cognitive	Slower choice reaction time with binge drinking episodes.

*AA, Absolute Alcohol; ADBB, Alarm Distress Baby Scale; AQS, Attachment Q-Set; ASQ, Ages and Stages Questionnaire; AUDIT, Alcohol Use Disorder Identification Test; BNBAS, Brazelton Neurobehavioral Assessment Screen; BRIEF, Behavioral Rating Inventory of Executive Function; BRS, Behavioral Rating Scales; BSF-R, Bayley Short Form-Revised BSID, Bayley Scales of Infant Development; CBCL, Child Behavior Checklist; CDIW, Communication Development Inventory—Words; CELF-P, Clinical evaluation of language fundamentals, Preschool version; CPT, Computerized Performance Task; DTS, The Difficult Temperament Scale; ELMS, Early Language Milestone Scale; F-BAS, Frequency-Binge Aggregate Score; DDST, Denver Developmental Screening Test, FIST, Flexible item selection task; FTII, Fagan Test of Infant Intelligence; GMDS, Griffiths Mental Developmental Scales; HOME, Home observation of the environment; IBR, Infant Behavior Record; IBQ, Infant Behavior Questionnaire; ITSC, Infant/Toddler Symptom Checklist; IQ, Intelligent Quotient; JSAIS = Junior South African Individual Scales; MABC, Movement Assessment Battery for Children; MAST, Michigan Alcoholism Screen Test; MCRS, Mother-Child Rating scales; MSAC, Maternal Substance Abuse Checklist; MSCA, McCarthy Scales of Children's Abilities; MFDD, Munich functional developmental diagnostics; NCT, Noncanonical commands test; NCATS, Nursing Child Assessment Teaching Scale; NRDLS, New Reynell Developmental Language Scales; NTB, Neurobehavioral test battery Oz, ounces; PBCL, Personal behavior checklist; PDS, Pictorial Depression Scale; pFAS, Partial fetal alcohol syndrome; RSA, Republic of South Africa; SB, Stanford-Binet; SFP, Still Face Procedure; SDQ, Strengths and Difficulties Questionnaire; SICD, Sequenced Inventory of Communication Development; TAC, Teller Acuity Cards; TI, Tapping Inhibition; TPT, Tactual performance test; UK, United Kingdom; USA, United States of America; VABS, Vineland Adaptive Behavior Scales; VRM, Visual Recognition Memory; WFMSB, Wisconsin Fine Motor Steadiness Battery; Gross motor scale; WPSSI, Wechsler Preschool and Primary Scales of Intelligence*.

Regarding the age groups investigated in studies, the neonatal age-group was the area with least investigation (*n* = 7), followed by toddlers (i.e., 24–47 months; *n* = 10). The majority of studies (*n* = 32) investigated outcomes in infants (i.e., 4–23 months) and pre-school aged children (i.e., 48–66 months; *n* = 27). Most studies assessed global developmental outcomes using assessment tools that combine outcomes across several developmental domains to provide a general indication of development. The most frequently used assessment tool was the Bayley Scales of Infant and Toddler Development infants (22, 41, 47, 51, 57–59, 62, 64–66, 68–71, 77, 79). A number of other tools were also used in studies in this review and are listed in Table 1.

Compared to studies focusing on global developmental outcomes, less research focused on the effects of prenatal alcohol exposure on specific domains such as motor (81, 84, 95), language (24, 74, 76, 77, 83), executive functions (88, 99, 102), and aspects of emotional and behavioral functioning (48, 49, 53, 56, 59, 60, 63, 75, 82, 89, 90, 92, 98, 100, 101, 103). A small body of research focused on specific functional outcomes of interest in executive function such as attention span (67), sustained attention (86), processing speed (52, 55, 109), and reaction time (54, 85, 104).

## Discussion

This review investigated the effects of prenatal alcohol exposure on developmental outcomes during early childhood. The following section will discuss summary findings from the literature on the general developmental outcomes and domain-specific effects of prenatal alcohol exposure on neonates, infants, toddlers, and preschool age children with the aim to highlight the gaps in our understanding and directions for future research.

### Effects of Prenatal Alcohol Exposure on Neonatal Outcomes

The fewest number of studies focused on the effects of prenatal alcohol exposure on neonates. Effects associated with abnormal motor and behavioral functioning amongst neonates with prenatal alcohol exposure include decreased arousal (42–45), decreased orientation (39, 40), decreased habituation (45), abnormal reflexes (38, 40), increased irritability (44), and decreased muscle tone (38). Although the majority of studies within this age group demonstrate that poorer outcomes are related to longer duration and greater dose of exposure (38–40, 42), it appears that negative outcomes such as poorer arousal and increased irritability can be detected with exposure as low as 1 drink per day (44).

Findings from apical assessments such as the BNBAS typically provide a general indication of atypical functioning, rather than a behavioral profile specific to the effects of prenatal substance exposure (110) and therefore have limitations in aiding clinicians' diagnosis of FASDs. Findings from this review do however indicate that the central nervous system deficits detectable in neonates may be predictive of delayed or atypical development later in infancy. For example, Brazelton Neurobehavioral Assessment Screen (BNBAS) scores measured by Coles, Smith (41) at 3 days predicted infants' mental and psychomotor development index scores on the BSID at 6 months. These findings suggest that the poor developmental outcomes associated with alcohol exposure may be identifiable in the first days and weeks of life before higher order cognitive functions emerge and postnatal environmental factors confound developmental functioning (45). The value of these neonatal studies in predicting cognitive, motor, and behavioral developmental outcomes later in childhood, support the crucial need for the use of a standardized screening protocol to help clinicians identify neonates who may have been exposed to alcohol prenatally and will thus benefit from closer monitoring of their development (8).

Relative to older infants and children, relatively little research has investigated the early neurobehavioral effects of prenatal alcohol exposure, and several research gaps remain. The majority of studies investigating outcomes in neonates drew samples from North American populations mostly consisting of middle-income participants. Further research drawing from a wider population base including a greater range of socio-economic and cultural environments is required to clarify the generalisability of the existing findings. Similarly, evidence on how outcomes identifiable during the neonatal period may predict functioning later in infancy in relation to alcohol exposure patterns remains limited (40). Furthermore, the actual mechanisms by which the effects translate into real life developmental and neurobehavioral outcomes are poorly understood and are compounded and confounded by the complexity of the CNS, rapid body change with time, and other factors.

### Effects of Prenatal Alcohol Exposure on Infant Development

Relative to other developmental stages, there is extensive literature on the effects of prenatal alcohol exposure in the infant age-group. The majority of findings from developmental assessments are suggestive of global developmental impairment (i.e., impairment to both cognitive and motor domains) and developmental delay amongst infants with prenatal alcohol exposure (17, 29, 41, 46, 47, 51, 57, 59, 66, 72, 73, 76). However, developmental assessments of children in this age group did not always find impairments to both cognitive and motor domains. Three studies utilizing the BSID for example only found lower MDI (cognitive) scores amongst infants, between 1 and 2 years of age, with prenatal alcohol exposure (64, 65, 68) while PDI (performance/motor) outcomes were non-significant when compared to unexposed controls. Four studies found marked motor delays, such as delayed sitting, standing, and walking in infants between 6 and 18 months (17, 46, 69, 72) with prenatal alcohol exposure compared to controls. These delays appear apparent amongst infants with longer duration (i.e., exposure throughout pregnancy) and heavier doses of prenatal alcohol exposure (69, 72), confirming known trends on the dose-response relationship between the amount and duration of prenatal alcohol exposure and the likelihood of motor delays.

Looking at specific domains, some investigators found evidence that poor language outcomes could be identified in infants as young as 6 months of age (51), while others described older infants with prenatal alcohol exposure having poorer comprehension and spoken language when compared to healthy controls (17, 68, 72). These investigations demonstrate that the poor language abilities in infants with prenatal alcohol exposure are present across social and linguistic contexts in the USA, Western Europe, and rural communities within South Africa and appear in studies with low (68) and heavier (17) doses of alcohol exposure. Studies tended to use different methods to assess language however, making comparisons on language development within this age band difficult.

Studies investigating outcomes in this age group also suggest that infants with prenatal alcohol exposure may display a wide range of emotional and behavioral deficits identified using observational self-report rating scales (22) and experimentally induced paradigms such as the still face procedure (56, 60). These deficits include poor emotional regulation (51), emotional withdrawal (53), few social monitoring behaviors (60) increased irritability (48, 63), difficult temperament (49), passive behavior and lack of social engagement (22, 59).

Prenatal alcohol exposed children at-risk for developmental delays, or who meet the diagnostic classification for FAS (17, 66), are those whose mothers consumed alcohol frequently, for prolonged periods, at moderate-to-heavy doses (46, 72), and binges (51). However, there is evidence that some functions related to information processing such as reaction time may be sensitive to much lower doses (i.e., doses equivalent to 1 drink per day) of exposure (54) and are observable at this early age. Other aspects of information processing such as fixation duration measured on a visual recognition memory paradigm (111) appear more apparent when infants are exposed to heavy frequent exposure (50, 55) as opposed to heavy infrequent exposure such as during occasional binge drinking (52). Thus, while the overall pattern of results is indicative of a dose-response relationship between prenatal alcohol exposure and developmental outcomes, research gaps remain in identifying whether there is a threshold level of effects associated maternal drinking during pregnancy (112).

### Effects of Prenatal Alcohol Exposure on Toddler Development

A number of studies investigated outcomes associated with prenatal alcohol exposure within this age group. Although the majority of studies demonstrate poorer cognitive scores in toddlers with prenatal alcohol exposure (65, 77, 113), developmental assessment findings on toddlers with prenatal alcohol exposure compared to their unexposed counterparts are not entirely consistent in the literature (see 48, 114). Findings demonstrating no effects of prenatal alcohol exposure on developmental outcomes were more common in high income countries where children are exposed to fewer risk factors (114) than in settings where FASDs are prevalent (12). Alcohol consumption patterns in these contexts are also less detrimental to fetal health than in contexts where heavy, binge drinking on weekends is common (115, 116).

Conducting studies on 2–3 year olds when key and more complex domains start developing broadens the range of deficits that can be assessed in children with prenatal alcohol exposure. Identifying developmental delays related to prenatal alcohol exposure amongst children during this period may allow clinicians to identify children who are “at risk” and may benefit from interventions targeted the specific developmental outcomes that they may struggle with at school age. A greater proportion of literature on 2–3 years olds report domain specific findings such as gross motor (81) fine motor (76, 81), and behavioral problems such as hyperactivity, difficulty managing behavior, and tantrums (78, 82) in 3 year olds with prenatal alcohol exposure. Faden and Graubard (78) found that behavioral problems in their sample were present despite there being no other effects of prenatal alcohol exposure on children's developmental indices. It is possible that carer-observed behavioral problems associated with prenatal alcohol exposure may be easier to identify than overall developmental problems which are in comparison less apparent and may require clinical expertise to formally assess.

Regarding the impact of prenatal alcohol syndrome on language abilities, evidence is mixed and only two studies demonstrate that 2–3 year old children with prenatal alcohol exposure perform poorly on language tasks measuring expressive and comprehensive language abilities (77, 113). It is possible that these effects are dose specific where lower levels of alcohol exposure are more subtle and difficult to detect on language tests amongst this age group as results from studies on toddlers suggest that language delays are not apparent in children with lower levels of exposure (< 1 drink on a weekly basis or less) (24) but are more evident in children with binge pattern exposure (24) and longer durations of exposure (77).

Some research also demonstrated poorer language outcomes in both controls and alcohol exposed infants from low socioeconomic environments, suggesting that poorer language outcomes may result from post-natal environmental factors rather than the teratogenic effects of alcohol on language functioning (76). There is evidence on the strong negative effect of low maternal education and SES in South African cohorts in older children (25). Other research suggests that language deficits in children with prenatal alcohol exposure is in keeping with children's general intellectual functioning, and the effects of prenatal alcohol exposure on language are no longer evident when IQ is controlled (83). Since higher functions have not yet emerged during these early years, research conducted on young children may not be able to establish whether the impairments amongst children with prenatal alcohol exposure are a product of generalized cognitive difficulties (measured by Intelligent Quotient testing) or occur independent of children's overall IQ (31). Findings from child data does, however does provide guidance to researchers interested in the trajectories of prenatal alcohol exposure regarding development domains that should receive focus at later time-points.

Regardless of the underlying nature of the language difficulties amongst children with FASDs, language impairments amongst children exposed to alcohol prenatally are likely to impact other aspects of their functioning such as their working memory (117) abilities and their social and interpersonal abilities (118). Impaired language abilities may be especially significant in inhibiting the adaptive functioning of children with prenatal alcohol exposure during the pre-school period where children are prepared for the transition to school. Thus, language functioning amongst young children with prenatal alcohol exposure remains an important area of investigation.

### Effects of Prenatal Alcohol Exposure on Preschool Age Development

A substantial body of literature has investigated a wide range of outcomes associated with prenatal alcohol exposure amongst preschool age children. Findings on general development in 4–5 year old children with prenatal alcohol exposure are variable. Whilst some studies indicate lower IQ scores on the WPSSI in children with prenatal alcohol exposure (87, 89), an equal number of studies did not find any differences in IQ scores between those with and those without prenatal alcohol exposure (61, 97). Falgreen Eriksen, Mortensen (97), however, found that risk for poor performance was dose-dependent and children with higher levels of exposure (i.e., mothers who consumed 9 or more drinks per week) was associated with lower IQ scores. Investigations that used the McCarthy Scales of Children's Abilities appear more consistent and demonstrate lower scores in the cognitive index (91) and overall scores (47) in children prenatally exposed to alcohol and children diagnosed with FAS (103).

Regarding the effects of prenatal alcohol exposure on motor functioning, some studies suggest that social drinking (i.e., consuming 1 drink per day) (84), but not isolated episodes of binge drinking (i.e., consuming 5 or more drinks per occasion), (95) is associated with poorer gross and fine motor abilities. Deficits in attention, social and behavioral and executive functioning that are well-established in older children with prenatal alcohol exposure also appear prominent amongst preschool aged children with prenatal alcohol exposure (119, 120). Studies that investigated social and behavior functioning demonstrate greater negative affect (92, 100), emotional and conduct problems (101), and greater frequency of insecure attachment (100) amongst children with prenatal alcohol exposure and a greater frequency of behavioral problems in children with FAS (103).

Emerging executive functioning difficulties in children with prenatal alcohol exposure include lack of inhibition [measured in a tapping task (88), cognitive flexibility, and delay of gratification (102)]. These difficulties along with poorer sustained attention (85) and choice reaction time (104) amongst children with prenatal alcohol exposure may play a key role in the adaptive functioning difficulties evident in children and adolescents with prenatal alcohol exposure (121, 122). Cognitive and behavioral difficulties during 4–5 years of age are a key limitation in children's ability to cope with the demands of the pre-school curriculum, which in turn negatively impacts their ability to cope with the cognitive and social demands of the schooling environment. The presence of young children with FASDs and cognitive and behavioral difficulties are especially challenging in resource-limited regions with high prevalence rates of FASDs that are heavily burdened by the effects of impoverished conditions and have (17). It is imperative that early interventions to ameliorate the effects of prenatal alcohol exposure are suitable and feasible for low-income contexts where the majority of cases of prenatal alcohol exposure are present.

### Longitudinal Investigations on the Effects of Prenatal Alcohol Exposure on Early Development

A number of papers within this review comprised of cross-sectional analyses from longitudinal studies (17, 44, 51, 123, 124). Longitudinal studies have been conducted in various population groups including both middle class (38) and low income groups (86, 125) These studies highlight the enduring effects of prenatal alcohol exposure through the duration of childhood and provide key information on how socio economic factors may interact with the effects of alcohol exposure in impacting children's developmental trajectories (29). They also highlight the complexities in identifying the effects of prenatal alcohol exposure over the developmental trajectory and the need for early intervention that mitigates the negative enduring effects of prenatal alcohol exposure. Given that the bulk of longitudinal investigation have been conducted in North American contexts, relatively less is known on the longitudinal outcomes associated with prenatal alcohol exposure in lower-middle income contexts.

### Limitations and Directions for Future Research

Studies in this review demonstrate clear global impairment related to FAS (17, 29, 66, 102, 103) but several inconsistencies in research evidence regarding the global and specific effects of low to moderate alcohol exposure on early development (34, 58, 61, 71, 74, 80, 86, 95). In order to gain clarity on the effects of light drinking during pregnancy on child developmental outcomes, further research should aim to address the potential methodological challenges that may contribute to these inconsistent findings on the effects of low doses of prenatal alcohol exposure on child development.

Findings indicative of null effects related to prenatal alcohol exposure appear prevalent in studies where participants report low levels of alcohol exposure (61, 79). It is possible that the developmental tools commonly used to identify global developmental delays common in children with heavier exposure/diagnoses of FAS are less sensitive in detecting the specific effects of prenatal alcohol exposure commonly associated with lower levels of exposure (69, 126). Given that some domains are sensitive to low doses of exposure (69), further research should investigate the suitability of tools that provide a more detailed assessment on aspects of functioning (such as behavior and language) that are less easily detected on general functioning tests (78).

In population-based studies where negative outcomes associated with prenatal alcohol exposure have been reported, studies have frequently reported on a small proportion of participants with moderate to severe prenatal alcohol exposure and are thus often unable to reliably measure the outcomes associated with various doses of alcohol exposure. Given that drinking during pregnancy is heavily stigmatized, participants within these studies may be likely to underreport their alcohol consumption. In addition, studies often lack detailed information on the potential confounding effects of sociodemographic and psychosocial factors that play a key role when investigating the outcomes related to prenatal alcohol exposure. For example, further investigation suggests that the better neurodevelopmental outcomes in children with prenatal alcohol exposure compared to controls in the sample assessed by Bay and colleagues (94) may likely be due to increased socioeconomic protective factors (e.g., higher maternal education levels) amongst the prenatal alcohol exposure group (127). Better understanding the contribution of the role of confounding factors on the outcomes related to prenatal alcohol exposure may help understand some of the inconsistencies in research studies published to date.

The pattern of some inconsistencies in the findings described in this review suggest that outcomes related to prenatal alcohol exposure may be more apparent at particular stages of early development. For example, Fried and colleagues research demonstrate that findings evident in their sample at 36 months were no longer evident at 48 or 60 months (79, 93). In addition the inconsistent findings on the effects of prenatal alcohol exposure on IQ findings amongst preschool age children may suggest that the negative effects of prenatal alcohol exposure on IQ functioning are more identifiable when children reach school age when cognitive demands are higher (128). Furthermore, longitudinal findings also suggest that developments tools such as the BSID may be less able to detect less apparent effects of prenatal alcohol exposure at certain developmental periods (126). Similarly, given that decreased IQ scores amongst school-aged children with prenatal alcohol exposure in comparison to healthy controls are common, current IQ tools may be limited in establishing children's IQ abilities during the pre-school years (21). These studies suggests the importance of investigating the timing during development at which specific developmental abnormalities can be identified and highlight the importance of understanding the trajectory of the developmental outcomes related to prenatal alcohol exposure.

Research that investigates the longitudinal trajectory of prenatal alcohol exposure demonstrate that the negative effects of prenatal alcohol exposure tend to endure as cognitive demands increase over childhood (123, 124). Recent experimental findings in rodents demonstrating the persistent effects of binge-type alcohol exposure on pyramidal neurons in the somatosensory cortex support these findings (129). Deficits in developmental domains identified earlier in infancy may also compound the development of other developmental domains. For example, the poor attention span present during infancy (55, 73) impacts the development of early executive functioning abilities that often require sustained attention as a prerequisite (102). The developmental effects of prenatal alcohol exposure may be exacerbated by risk factors related to low socio economic status (e.g., poor nutrition, lack of stimulation, exposure to violence) (17, 29). These risk factors related to the postnatal environment however, may mask the effects of the teratogenic influences of alcohol exposures and thus add to the methodological challenges in investigating the effects of prenatal alcohol exposure on developmental outcomes. The increased occurrence of null findings in older ages groups in this review supports the possibility of socioeconomic influences masking the differences between children prenatally exposed to alcohol and matched controls.

Future longitudinal research is warranted to address the paucity of research conducted in low income settings to improve our understanding of the interaction between prenatal alcohol exposure and potential socioeconomic factors on developmental outcomes (130). Understanding how these outcomes manifest during early childhood will be crucial in designing interventions that are feasible in low-income settings and able to minimize the enduring effects of prenatal alcohol exposure on adaptive functioning throughout childhood (121, 122). Further longitudinal investigations that investigates the early developmental trajectories outcomes related to prenatal alcohol may also provide an important direction for research that aims to delineate a cognitive profile of FASDs (31, 131).

In comparison to knowledge on the cognitive and behavioral developmental outcomes, there is less knowledge on other aspects of development that are influenced by maternal alcohol consumption during pregnancy. Exposure to alcohol *in-utero* affects multiple organs and children with prenatal alcohol exposure are at increased risk for developing comorbid conditions including hearing impairments (132), visual impairments (133), and sensory processing disorders (134). These disorders further hinder children's developmental potential and results in difficulties coping with the school environment (135), thus further research on these comorbid conditions will be highly beneficial to improving long-term outcomes amongst children with FASDs. In addition while some studies within this review reported growth outcomes amongst their samples (17, 29, 52), the majority of studies did not include child growth outcomes in their analyses of developmental outcomes. Since growth restrictions may not always be present in children with prenatal alcohol exposure (136) and growth restrictions in young children may be a result of many factors other than prenatal alcohol exposure (e.g., nutrition status and genetics), poorer growth outcomes alone may be insufficient to identify children who may have been exposed to alcohol *in-utero*. Regardless, understanding the growth trajectory of children exposed to alcohol *in-utero* may be of clinical value as findings demonstrate that growth trajectories may serve as an important biomarker to identify children at greatest risk for the cognitive deficits associated with prenatal alcohol exposure (51, 137).

Another key avenue for further research is to explore where alcohol consumption during pregnancy affects males and females differently. Studies within this review suggests that girls exposed to alcohol *in-utero* are more vulnerable to the effects of prenatal alcohol exposure (82), and that patterns of stress reactivity differ between girls and boys with prenatal alcohol exposure (56). Although several animal studies have explored the effects of prenatal alcohol exposure on males and females, this area of research has been less investigated in humans (138, 139). Given that these findings have implications in the diagnosis and intervention of FASD's, further research should aim to clarify and further understand these findings.

Although this review followed a systematic approach, it has several methodological limitations. First, several studies within this review (e.g., birth cohort studies) drew their data from the same pool of participants and findings from those samples may be repeated and overrepresented within this review. Although evidence from longitudinal studies is valuable in tracking the developmental manifestations of prenatal alcohol exposure throughout development, a significant proportion of evidence on the effects of prenatal alcohol exposure within this review comes from investigations with potential overlap between samples. Second, heterogeneity in the design and methodologies of studies included in this review limits our ability to interpret results across cohorts and studies and in being able to fully interpret inconsistencies this review. For example, due to cultural variations in early language acquisitions and challenges in interpreting outcomes on translation tests, findings on the effects of prenatal alcohol exposure on language outcomes, are difficult to compare and interpret (140). In comparison to neonatal studies, studies on infants and older age groups have greater heterogeneity due to a wider range of methodologies used to assess developmental outcomes. Finally, current findings are also difficult to interpret as studies use a wide range of systems to classify alcohol exposure. Greater standardization in the classification of alcohol exposure may aid the understanding of the developmental outcomes associated with prenatal alcohol exposure in infants, particularly in ascertaining the effects associated with low and moderate doses of alcohol use during pregnancy. The use of biomarkers may provide an objective means to identify infants exposed to lower doses alcohol *in-utero* (141).

In sum, studies within this review highlight the complex relationship between dose of prenatal alcohol exposure and its various associated developmental outcomes. This review shows that the cognitive and behavioral effects of prenatal alcohol exposure seen in older age groups are apparent during the early childhood period. The specific outcomes of lower to moderate doses prenatal alcohol exposure on specific outcomes such as early language development remains poorly understood. Further research should aim to clarify the more subtle manifestations of prenatal alcohol exposure on early development as well as core early deficits which predict later functional outcomes.

## Author Contributions

SS selected the literature, extracted data, and compiled the first draft of the manuscript. EE assisted with the literature search, data extraction, and provided critical review of the manuscript. KD conceptualized the review and provided critical review of the manuscript. CA and DS provided critical review of manuscript drafts. All authors approved the final draft.

### Conflict of Interest Statement

The authors declare that the research was conducted in the absence of any commercial or financial relationships that could be construed as a potential conflict of interest.
